# COVID-19 vaccine acceptance rates and predictors among the Egyptian general population and Healthcare workers, the intersectionality of age and other factors

**DOI:** 10.1038/s41598-022-23825-2

**Published:** 2022-11-18

**Authors:** Engy Mohamed El-Ghitany, Ayat Ashour, Eman A. Omran, Azza Galal Farghaly, Mahmoud A. Hassaan, Nashwa Fawzy Abd El-Moez Azzam

**Affiliations:** 1 Department of Tropical Health, High Institute of Public Health, Alexandria University, Alexandria, 21526 Egypt; 2 Department of Family Health, High Institute of Public Health, Alexandria University, Alexandria, 21526 Egypt; 3 Department of Microbiology, High Institute of Public Health, Alexandria University, Alexandria, 21526 Egypt; 4 Institute of Graduate Studies and Research, Alexandria University, Alexandria, Egypt

**Keywords:** Health care, Medical research

## Abstract

The promise of COVID-19 vaccines in ending the pandemic can only be achieved by overcoming the challenge of vaccine refusal. Healthcare workers (HCWs) are the trusted advisors of vaccination decisions. Recommendations for vaccinating children against COVID-19 are recently gaining more public health attention due to the role of children in disease transmission and associated morbidities. Vaccination is one of the first medical decisions parents or guardians make on behalf of their children. To investigate the determinants associated with vaccine acceptability among the general population through a direct interview questionnaire and assess guardians’ views towards childhood COVID-19 vaccinations. This cross-sectional study included 2919 participants A pre-designed structured questionnaire about COVID-19 vaccination acceptability was completed by trained interviewers and interviewing the participants or their guardians (for those below 18 years old). Nearly two-thirds of participants (66.5%) accepted vaccination, 20.2% were refusing and 13.3% were hesitant. Most participants who were guardians of children below 12 years and from 13 to 17 years reported that they would accept vaccination of their children (72.5% and 70.5%, respectively). The acceptance rate among HCWs was 58.2%. The main reasons beyond vaccine refusal were mistrust of vaccine efficacy (39.5%) and having concerns regarding vaccine safety (38.8%). In a multivariable regression model, being male (OR 1.362, 95% CI 1.082–1.714, p = 0.008) resident in rural area (OR 1.796, 95% CI 1.435–2.247, p = 0.000), and lower education (OR 1.245, 95% CI 1.018–1.523, p = 0.033) were associated with an increased acceptance to be vaccinated. The acceptance rate for vaccinating children reported among their guardians was higher than adults for themselves. Extremes of age showed higher vaccine acceptance compared to young adults. Upper Egypt governorates (Faiyum and Giza) were outpacing Lower Egypt governorates in vaccination acceptance rates.

## Introduction

Vaccination reduced the mortality rates of major communicable diseases over decades. Correspondingly, mass coronavirus disease-2019 (COVID-19) vaccination ultimately might end the COVID-19 pandemic by obtaining herd-immunity level coverage, enabling communities to return to their pre-COVID-19 state^1^.

The World Health Organization (WHO) reported COVID-19 vaccine delivery challenges due to the significant roadblock, vaccine acceptance^2^. It is defined as “the degree to which individual question, accept, or refuse vaccination.” It is an essential indicator for vaccine uptake rate (vaccination uptake = access + acceptance), and consequently, vaccine distribution success^3^.

Attitude toward the vaccines is dynamic and considerably changes over time as it is affected by complex personal factors, complacency (do not perceive a need for a vaccine, do not value vaccination), convenience (access to vaccines), effectiveness, side effects, novel techniques of vaccines and risk of COVID-19 exposure^4^. Healthcare workers (HCWs) are strong advocates for better dissemination of vaccine knowledge and boosting vaccine acceptance among the general population^5^.

Acceptance of COVID-19 vaccines among samples of the adult Egyptian population and HCWs was investigated in certain governorates through online surveys^6–9^. Despite the easiness of performing these online surveys (especially during the COVID-19 pandemic), they are known to have accessibility issues and selection bias compared to the interview questionnaire due to the under-representation of some population sectors such as children, the elderly, and illiterates^10^. This highlights the importance of investigating vaccine acceptance determinants via interview questionnaire across different governorates of Egypt. Moreover, to the best of our knowledge, there is scarce information on the Egyptian parents’ or guardians’ acceptability of COVID-19 vaccines to their children (below 18 years). The success of the future COVID-19 vaccination program for children will depend on guardians’ views towards childhood vaccinations. Exploring the acceptance rate and their predictors would support establishing new policies to promote vaccine uptake.

A global vaccine survey across several countries recognized insufficient knowledge about the vaccines, misinformation from social media, little efficacy and serious adverse effects causing death, manufacturers do not disclose adverse effects of vaccines, and political affiliation as the perceived barriers for accepting of COVID-19 vaccination^11–13^.

The aim of the present study was to determine the rate of vaccine acceptance and shed light on its predictors among the Egyptian population for themselves or their children across different governorates and among HCWs by direct interview questionnaire.

## Methods

A cross-sectional survey-based study was conducted between January and June 2021. This period coincided with the second and third waves of the COVID-19 pandemic in Egypt. According to the Egyptian Ministry of Health and Population, the second pandemic wave of COVID-19 had stricken Egypt from November 2020 till January 2021, and the third wave started from March 2021. At that time, vaccines for COVID-19 were reserved primarily for HCWs and high-risk groups of the general population. Only two vaccine types were available, namely, Sinopharm BBIBP and Oxford/AstraZeneca COVID-19 vaccine (AZD1222).

### Study population

This study was a part of a project which enrolled 2919 participants (2276 participants from the community and 643 HCWs from 39 hospitals across Egypt). The study was performed across ten Egyptian governorates, eight from Lower Egypt (Alexandria, Monufia, Cairo, Suez, Qalyubia, Dakahlia, Beheria, Kafr Elsheikh), and two from Upper Egypt (Faiyum and Giza). All governorates, settings and study participants were selected randomly. The sampling techniques were described elsewhere^14^.

### Data collection

A pre-designed structured questionnaire was completed by trained interviewers interviewing the participants or their guardians (for those below 18 years old). The study questionnaire was comprehensive but easy to understand. It comprised multiple sections with 90 items; one section included all general demographic information; age, sex, residence, occupation, education, lifestyle behaviors, and anthropometric data. The height and weight of all participants were measured to calculate their Body Mass Index (BMI). Standard adult BMI groupings were applied: underweight (BMI < 18.5), normal weight (BMI 18.5–24.9), overweight (BMI 25.0–29.9), and obese (BMI ≥ 30). Children's BMI classification was as follows; underweight (< 5th percentile), normal weight (5th to < 85th percentile), and overweight/ Obese (> 85th percentile). Associated comorbidities were recorded. Another section of the questionnaire comprised questions about contact with COVID-19 patients, history of COVID-19 infection, nature, and severity of symptoms. Some work setting data of HCWs were included in the questionnaire sheet, such as job category (physicians, nurses, pharmacist, etc.) type of hospital (COVID-19 isolation hospital or not). Departments of HCWs were categorized as being high-risk departments; intensive care units, emergency room, internal medicine wards, radiology, laboratory, or outpatient clinics) versus low-risk departments (others).

Subsequently, participants were asked about their acceptance to receive a potential COVID-19 vaccine by the following questions: “Do you agree to receive COVID-19 vaccine?” Answer options included “Yes,” “No,” and “Not sure.”, and if the answer was “no”, an open question was asked to assess the reasons for vaccine rejection “what is the cause of refusal?” The general acceptance of COVID-19 vaccine was the primary outcome variable of the study. It was categorized as accepting (Yes), refusing (No), and hesitant (Not sure).

To delineate spatial pattern of vaccine acceptance in the study area, the collected data were mapped using ArcGIS (ver. 10.8) (ESRI 2020) and two thematic maps were produced representing the vaccine acceptance and vaccine hesitancy in different governorates. Such mapping can highlight spatial variations in vaccine acceptance in the study area, which can be further justified by prevailing socioeconomic and cultural factors^15^.

### Statistical analysis

Data were fed to statistical software IBM SPSS version 22 (SPSS, Inc. Chicago, IL). Quantitative variables were expressed by the mean (X̅) and standard deviation (SD), while categorical variables by absolute and relative frequency. Descriptive analysis based on frequency and percent distribution was done for all categorical variables, including socio-demographic and behavioral data. Cross-tabulation and Chi-squared (χ^2^) test were used to analyze the associations between accepting the vaccine and the characteristics of participants. All statistical analyses were done using two-tailed tests. *A P*-value less than 0.05 was considered statistically significant. All questionnaire data was assessed in univariate analysis. Multiple logistic regression analysis was conducted to assess the significant independent predictors of COVID-19 vaccines’ acceptability with 95% confidence intervals (95% CI). For handling the missing data (where “not applicable” is the response), the model was conducted only on adult sample (n = 2352) and occupation related variables (e.g., type of the hospital, working hours) were removed from the model. The dependent variable was set as a dichotomous variable coded as “1” if the participant accept to take the vaccine, and “zero” otherwise (Refuse or hesitant).

### Ethics approval

The study was conducted in compliance with the Helsinki Declaration and was approved by the Institutional Review Board (IRB) Committee, Faculty of Medicine, Alexandria University; IRB number: 00012098—FWA number: 00018699, serial number: 0305136. Anonymity and confidentiality of participants were confirmed and written informed consent was obtained from each participant. For young participant less than 18 years, informed consent from a parent and/or legal guardian for study participation was obtained.

### Consent to participate

Written informed consent was obtained from all participants.

## Results

A total of 2919 participants completed the study questionnaire. Their age ranged from 2 to 90 years (mean = 37.07, SD 17.944 years). Most of them were in the age group of 25–39 years (820; 28.1%), followed by those aged from 40 to 49 years (533; 18.3%), and a minority of them were in the age group of 70–90 years (79; 2.7%). 567 (19.4%) were below 18 years. The majority of participants were women (1675; 57.4%), urban residents (2029; 69.5%), and married (76.5%). Most study participants reported having a university degree or higher (44.4%). Only 823 (28.2%) of participants had associated comorbidities. 43.1% of HCWs were physicians, 17.9% were nurses, and the remaining were technicians, pharmacists, and others.

**Figure 1:**
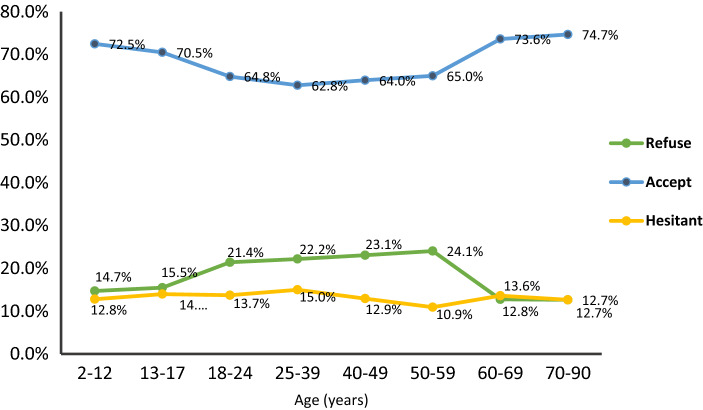
Vaccine acceptance in different age groups.

Participants demonstrated an overall 66.5% (1940/2919) acceptance rate to get a COVID-19 vaccine for themselves. Most participants who were guardians of children below 12 years and from 13 to 17 years reported that they would accept vaccination of their children (72.5% and 70.5%, respectively). The highest acceptability rate was among elderly group (70–90 years) (74.7%) (*p* = 0.001) (Fig. 1).

Males represented a higher percentage among the vaccine acceptance group compared to those who refused (45.1% vs. 38.5%, *p* = 0.001). Also, rural residence had significantly a higher frequency among the acceptance group compared the refusal one (35.6% vs. 18.3%, *p* = 0.000). Neither pre-existing comorbidities nor history of travel during the last six months or body weight were statistically associated with vaccine acceptability. Smokers constituted a higher proportion among the acceptance group compared to the refusal group (28.6% vs. 13.2%; *p* = 0.003) (Table 1).

**Table 1:** Factors associated with COVID-19 vaccine acceptance (n = 2919).

	Willing to receive COVID-19 vaccine						p-value
	No (refuse) (n = 590)		Yes (accept) (n = 1940)		Not sure (hesitant) (n = 389)		
	No	%	No	%	No	%	
**Gender**							0.001*
Male	227	38.5%	874	45.1%	143	36.8%	
Female	363	61.5%	1066	54.9%	246	63.2%	
**Residence**							0.000*
Urban	482	81.7%	1249	64.4%	298	76.6%	
Rural	108	18.3%	691	35.6%	91	23.4%	
**Comorbidities**							0.633
No	424	71.9%	1385	71.4%	287	73.8%	
Yes	166	28.1%	555	28.6%	102	26.2%	
**BMI**							0.210
Normal weight	154	26.1%	504	26.0%	96	24.7%	
Underweight	11	1.9%	24	1.2%	11	2.8%	
Overweight/ obese	425	72.0%	1412	72.8%	282	72.5%	
**Smoking status**							0.003*
No	512	86.8%	1385	71.4%	287	73.8%	
Yes	78	13.2%	555	28.6%	102	26.2%	
**Exercise**							0.096
No	104	17.6%	297	15.3%	75	19.3%	
Yes	486	82.4%	1643	84.7%	314	80.7%	
**History of travel abroad within the last six months**							0.676
No	587	99.5%	1923	99.1%	386	99.2%	
Yes	3	0.5%	17	0.9%	3	0.8%	

**Figure 2:**
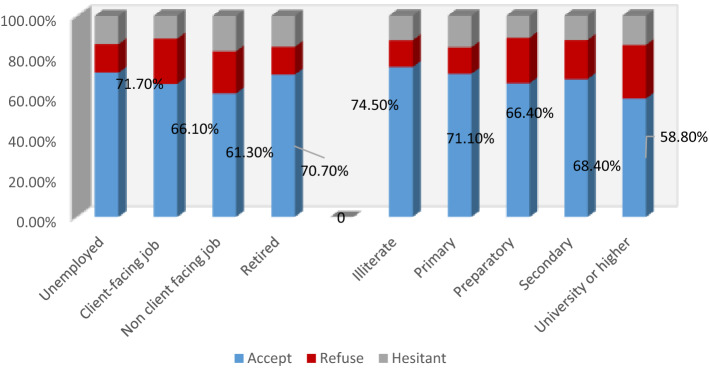
The acceptance of COVID-19 vaccines concerning occupation status and educational level.

Surprisingly, a significantly higher percentage of illiterates were accepting COVID-19 vaccination (74.5%; *p* = 0.000), followed by primary education (71.1%) and secondary education (68.4%). Regarding occupation, the highest acceptability was among unemployed (71.7%) and retired participants (70.7%), followed by those working at client-facing jobs (66.1%) (*p* = 0.000) (Fig. 2).

**Figure 3:**
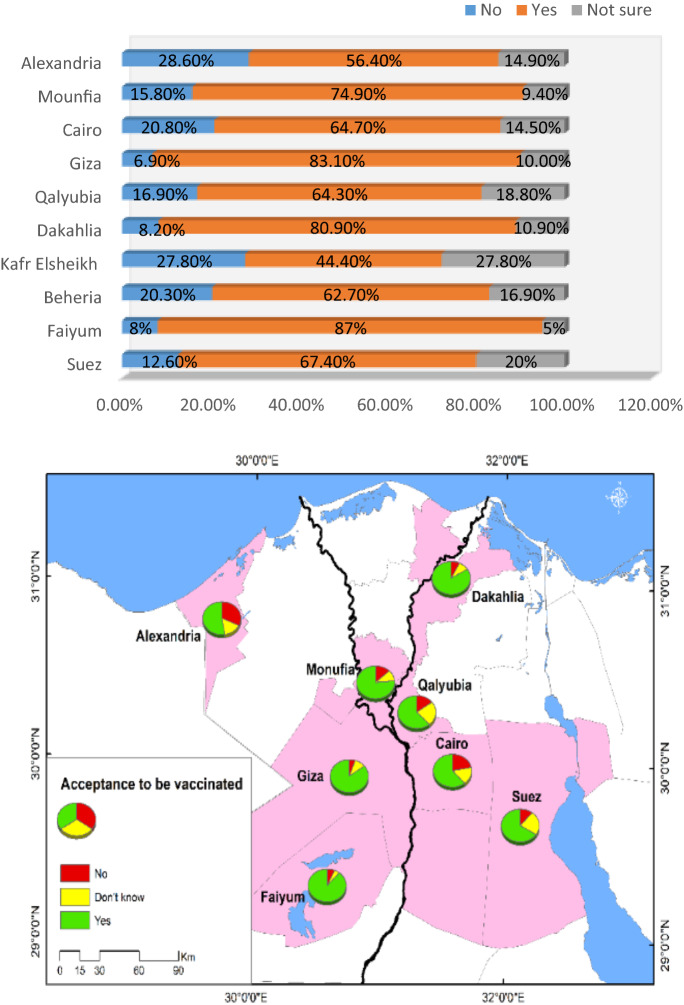
Vaccine acceptance in different Egyptian governorates.

As regards geographical distribution, residents of Faiyum Governorate were the most accepting of COVID-19 vaccination (87%), while those from Kafr Elsheikh Governorate were the least (44.4%) (Fig. 3).

Table 2 illustrates the acceptability of COVID-19 vaccines among participants according to COVID-19 associated factors. Out of all participants, 373 (12.8%) reported a previous history of COVID-19 infection. Their contribution to the refusal rate was higher than that to the acceptance rate (16.4% vs. 11.4%; *p* = 0.004). Those who reported having more than five symptoms of COVID-19 infection were less accepting to be vaccinated than those with no one or two symptoms (*p* = 0.004). Almost two-thirds (66.4%) of those who accepted to receive the vaccine did not report a history of contact with COVID-19 patients compared to approximately half (50.8%) of those who refused (*p* = 0.000).

**Table 2:** Relation between vaccine acceptance and COVID-19 associated factors (n = 2919).

	Willing to receive COVID-19 vaccine						P-value
	No (refused) (n = 590)		Yes (accept) (n = 1940)		Not sure (hesitant) (n = 389)		
	No	%	No	%	No	%	
**Contact with COVID-19 patient**							0.000*
No	300	50.8%	1288	66.4%	242	62.2%	
Yes	290	49.2%	652	33.6%	147	37.8%	
**History of COVID-19 infection**							0.004*
Never	493	83.6%	1719	88.6%	334	85.9%	
Yes (once or more)	97	16.4%	221	11.4%	55	14.1%	
**Number of reported COVID symptoms**							0.004*
0	172	29.2%	690	35.6%	142	36.5%	
1–2	160	27.1%	562	29.0%	110	28.3%	
3–4	121	20.5%	369	19.0%	71	18.3%	
5+	137	23.2%	319	16.4%	66	17.0%	

**Figure 4:**
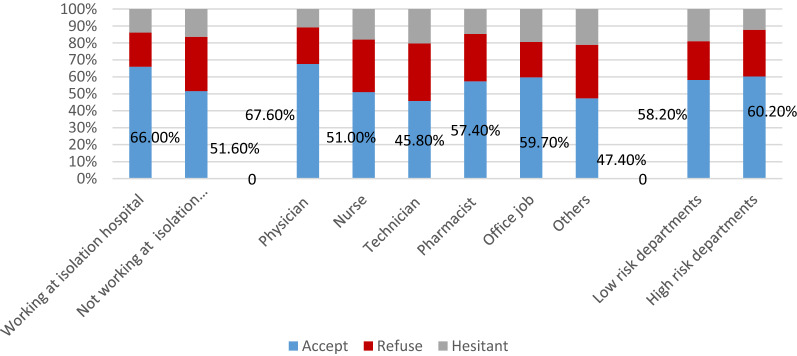
The acceptance of COVID-19 vaccines among HCWs according to job task, type of hospital and department.

Among HCWs, the acceptance rate was 58.2%. Physicians were the most accepting group (67.6%), whereas technicians were the least (45.8%) (*p* = 0.062). Higher acceptability was among those working in COVID-19 isolation hospitals (66% vs 51.6%; *p* = 0.001), and at high-risk departments (60.2% vs 58.2%; *p* = 0.069) (Fig. 4).

Analysis of the predictors of vaccine acceptance among adults is shown in Table 3. Results of multivariate analysis showed that significant independent predictors of vaccine acceptance were being male (OR 1.362, 95% CI 1.082–1.714, *p* = 0.008), rural residents (OR 1.796, 95% CI 1.435–2.247, *p* = 0.000), lower education (below university) (OR 1.245, 95% CI 1.018–1.523, *p* = 0.033), not working (OR 2.101, 95% CI 1.344–3.284, *p* = 0.001), working in client facing jobs (OR 1.560, 95% CI 1.028–2.368, *p* = 0.037), and practicing exercise (OR 1.278, 95% CI 1.011–1.617,* p* = 0.041).

**Table 3:** Predictors of vaccine acceptance among adult Egyptian participants (n = 2352).

	Crude OR (LL-UL)			P-value	Adjusted OR (LL-UL)			P-value
**Gender**								
Male	1.454 (1.221–1.732)			0.000*	1.362 (1.082–1.714)			.008*
Female (Ref.)								
**Age (years)**								
18–59 (Ref.)								
≥ 60	1.603 (1.227–2.094)			0.001*	1.264 (0.930–1.717)			0.134
**Residence**								
Urban (Ref.)								
Rural	2.106 (1.712–2.592)			0.000*	1.796 (1.435–2.247)			0.000*
**Crowding index**								
< 1 (Ref.)								
1- > 2	1.191 (1.002–1.416)			0.047*	1.130 (0.939–1.360)			0.195
2 +	0.979 (0.577–1.660)			0.937	0.955 (0.551–1.655)			0.869
**Educational level**								
Below university level	1.669 (1.407–1.980)			0.000*	1.245 (1.018–1.523)			0.033*
University graduate or above (Ref.)								
**Marital status**								
Not married (Ref.)								
Married	1.202 (0.988–1.462)			0.066	1.044 (0.847–1.289)			0.685
Comorbidities**								
No comorbidities (Ref.)								
Any comorbidities	1.172 (0.979–1.405)			0.085	1.131 (0.930–1.375)			0.217
**Occupation**								
Not working	1.642 (1.090–2.472)			0.018*	2.101 (1.344–3.284)			0.001*
Client facing job***	1.108 (0.749–1.637)			0.608	1.560 (1.028–2.368)			0.037*
Non-client facing job (Ref.)								
**Smoking status**								
No (Ref.)								
Yes	1.586 (1.258–2.000)			0.000*	1.259 (0.948–1.673)			0.112
**Exercise**								
No (Ref.)								
Yes	1.166 (0.935–1.455)			0.173	1.278 (1.011–1.617)			0.041*
**History of COVID-19 infection**								
Never	1.312 (1.044–1.650)			0.020*	1.091 (0.852–1.397)			0.490
Yes (Ref.)								
**Contact with COVID-19 patient**								
No	1.494 (1.259–1.772)			0.000*	1.043 (0.844–1.289)			0.698
Yes (Ref.)								
Applying (Practice) COVID protective measures***								
Not done (0)	1.253 (0.759–2.068)			0.378	1.165 (0.689–1.968)			0.569
Partially done(1–4)	1.110 (0.687–1.795)			0.669	1.010 (0.612–1.665)			0.970
Completely done (5) (Ref.)								
**History of travel abroad within the last six months**								
No (Ref.)								
Yes	1.826 (0.671–4.966)			0.238	2.583 (0.916–7.282)			0.073
**BMI**								
Normal (Ref.)								
Underweight	0.525 (0.187–1.472)			0.221	0.494 (0.166–1.470)			0.205
Overweight/obese	1.166 (0.947–1.436)			0.149	1.173 (0.938–1.467)			0.163

**Chronic morbidities include cardiac diseases, hypertension, diabetes mellitus, chronic renal and liver diseases.

***HCWs were included in the client facing jobs.

****Wear mask, wash hand, use soap for hand wash, use disinfectant, social distancing [each variable was binary(yes/no)].

**Figure 5:**
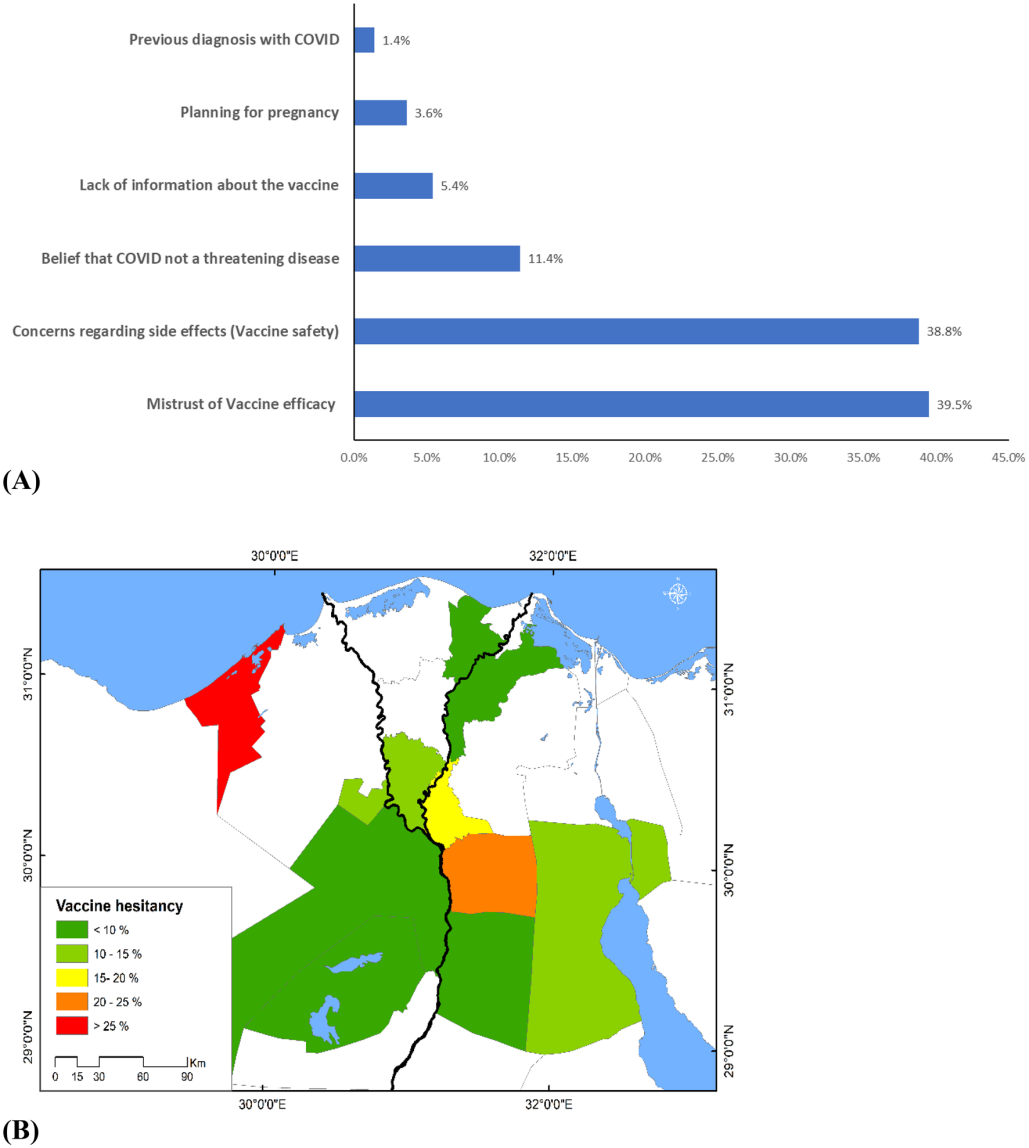
(**A**) Causes of vaccine refusal (**B**) Proportion of individuals who refused to receive COVID-19 vaccination across different governorates.

The commonest justifications of vaccine refusal were the mistrust of vaccine efficiency (39.5%), and concerns about the possible side effects (38.8%). In addition, a minority of participants argued that planning for pregnancy (3.6%) and previous diagnosis with COVID-19 infections (1.4%) were their causes of refusal (Fig. 5).

## Discussion

Egypt began its vaccination program in January 2021. Despite the tentative effectiveness and safety of the Chinese Sinopharm BIBP COVID-19 vaccine, the Egyptian Drug Authority (EDA), as in most Arab nations, had approved it for quarantine hospitals medical staff, followed by other medical teams, then high‐risk groups of the general population^16^. Hence, during this period, the vaccine acceptance rates might have been affected, where it reached only 8% among the general population^6^. As a part of the COVID‐19 Vaccines Global Access (COVAX) facility, a batch of Oxford/AstraZeneca (AZD1222) was obtained late in January 2021^17^. By the end of 2021, 70 million doses of different types of vaccines were delivered by UNICEF^18^. The availability of this wide variety of vaccines with different techniques might help raise vaccine acceptance rates. From April–May (2021), Elgendey and Abdelrahim reported an 88% acceptance rate among the general population through an online survey^19^. A lower percentage was recorded in the present study (66.5%), which can be considered a more representative result as it was delivered by interview questionnaires which are known to minimize the bias associated with online surveys such as multiple responses from participants and inaccurate self-reported demographic or characteristics information as well as selection bias. The acceptance rate was comparable at the regional level to a study among Saudi participants (64.7%)^20^. Much lower acceptance rate (25%) was recorded through survey that included four Arab countries (Jordan, Saudi Arabia, Lebanon, and Iraq)^21^.

The extraordinary rapid development of COVID-19 vaccines had put pressure on HCWs to practice their advisory role and transmit confidence to the public, as they are the most trusted source of information on vaccination. Individuals whose physicians recommend vaccination were more likely to be vaccinated^22^. Unfortunately, lower acceptance rates among HCWs were encountered in the current study (58.2%). The result agreed with the pooled acceptance rate of COVID-19 vaccine among HCWs around the world (55%)^23^. This observation might be attributed to that, just as the case with the general population, HCWs may have some misinformation about the vaccines, and that they were not feeling well informed about the techniques of the new vaccines, which might have led to concerns and mistrust of vaccine safety. The hopeful prospects are that HCWs' acceptance rate was higher than previous Egyptian studies (21–26%)^8,9^ and that the assessment of the HCWs intention was during the period of vaccines development and testing. Moreover, doctors, whom the community might have faith in, had the highest acceptance rate among other categories of HCWs. Therefore, HCWs' stance towards vaccines might change as more information on how COVID-19 vaccines are developed and tested becomes available. It should be noted that higher pooled acceptance rate (78%) among HCWs in China was reported which was explained by the availability of comprehensive scientific evidence and tremendous information related to COVID-19 and COVID-19 vaccines at an unprecedented pace which drive HCWs to become more aware of safety and effectiveness of different vaccines^24^.

Evidence suggests the importance of building the trust of guardians in COVID‐19 vaccines to be able to achieve children vaccination. According to WHO, an increasing number of vaccines had been authorized for emergency use for adolescent age group (12–17 years old) in some countries (messenger RNA (mRNA) vaccines; Pfizer-BioNTech (BNT162b2) and Moderna mRNA-1273). In November 2021, Pfizer-BioNTech (BNT162b2) had been approved for use in children aged 5–11 years, and Egypt's government announced to use it for vaccinating 12–15 years old children^25,26^. Sinopharm COVID-19 BBIBP and Sinovac-CoronaVac had been tried in children as young as age 3 years and the Chinese authorities approved them for the age of 3–17 years. Both mRNA vaccines' efficacy, immunogenicity, and safety were similar or higher compared to adults. In October 2021, the Global Advisory Committee on Vaccine Safety (GACVS) concluded that in all age groups, the benefits of mRNA COVID-19 vaccines outweigh the risks. Outbreaks of COVID-19 infections had been identified in secondary schools, summer camps and day-care centres. Children usually have fewer and milder symptoms compared to adults; however, they shed the virus through the respiratory tract and faeces to older and more vulnerable individuals. In addition, the indirect impact related to school closures and disruption of the educational services mandates children's vaccination^25^.

Limited literature had explored guardians’ perception towards vaccination of their children. Th acceptance rates of guardians were 64.0%, 72.6%, and 92.9% in low and middle income countries^27^. In the present study, most guardians of children below 12 years and from 13 to 17 years had a positive attitude towards vaccination for their children (72.5% and 70.5%, respectively). These findings correspond well with a study conducted in The United Arab Emirates, where the acceptance of the respondents to vaccinate their children reached 75.1%^28^. Also, similar result was recorded in England where 48.2% of participants accepted, and 40.9% were unsure but leaning towards vaccinating their children^29^. Therefore, information on the safety of vaccines for children must be communicated clearly to alleviate guardians’ concerns.

Several studies had reported the previous history of COVID-19 infection as a determinant of vaccine acceptance^30–32^. On the contrary, in the current study, the refusal rate among those with previous infection was higher than acceptance rate (16.4% vs. 11.4%; *p* = 0.004). This may be explained by their belief in protection. This conclusion was also arrived at by Alqudeimat et al., from Kuwait, where a lower acceptance rate was observed in those who believed that natural protective immunity had developed after natural infection^33^.

Almost two-thirds (66.4%) of those who accepted to receive the vaccine did not report a history of contact with COVID-19 patients compared to approximately half (50.8%) of those who refused (66.4% vs. 50.8%; *p* = 0.000) indicating risk perception of contracting COVID-19 infection and their confidence in the immunity conferred by COVID-19 vaccines. Physicians, especially those working at high-risk departments, were more accepting vaccination. However, acceptability was not statistically associated with the frequency of exposure to suspected cases/day. On the contrary, Fares et al. from Egypt outlined that those directly dealing with COVID-19 patients had 3 times higher odds of vaccine acceptance^8^.

Certain demographic and social determinants of vaccine acceptance should be considered to prevent disparities in vaccine uptake. Prior findings correspond well with the present study, where males represented a higher percentage among the vaccine acceptance group compared to those who refused^34,35^, which may be attributed to females’ panic of unanticipated side effects of vaccines on menstruation, pregnancy, and future pregnancy generations. On the contrary, Mohamed et al. from Malaysia recorded a higher acceptance rate among females^36^. Similar to the present study, acceptance of vaccination was reported to increase with age, which may be due to associated disabilities in older age groups^31,35^; however, a conflicting finding among younger age groups was reported in the United States, which may be due to different perceptions and beliefs of populations across countries^30^.

Unemployed and retired participants reported higher COVID-19 vaccine acceptance rates. A contradicting observation was recorded by Alqudeimat et al., where the retired group was the least accepting group^33^. Lower acceptability of COVID-19 vaccine was recorded among those with a university education or higher (58.8%) than other education levels. Nevertheless, national public health strategies should target college students and young adults who can transmit the infection to elderly risky groups owing to the sense of invulnerability. On the other hand, Patwary et al. from Bangladesh delineated that the highest acceptance rate was among those with a bachelor’s degree^37^. It should be noted that the direct interview questionnaire used in the present study facilitated the assessment of vaccine acceptance among illiterates (74.5%) by more engagement of the respondents who are not familiar with internet use and a better understanding of the questions, which improves data quality. To the best of our knowledge, all previously published similar studies were carried out using online surveys, highlighting our study's importance. High acceptance rate among low educated may be attributed to that highly educated persons may have more concerns about vaccine safety and long-term adverse reactions. They may use social media to search information on COVID-19 vaccination which had a significant impact on spreading fear and panic of vaccination. This finding agreed with those reported by Jabessa and Bekele from Ethiopia where low education was associated with greater willingness to take the COVID-19 vaccine^38^.

Health-related variables, such as long-standing comorbidities were not statistically associated with vaccine acceptance. Despite the growing evidence that COVID-19 vaccines exhibit short-term vaccination efficacy among obese subjects compared to those with normal body weight^39^, in our study, acceptance rate among obese was slightly higher than their refusal which was in accordance with a study from Kuwait^33^. That might be attributed to their fear of more grave complications related to COVID-19 in case they get infected. On the other hand, a study in Canada showed that obese people were more ambivalent about COVID-19 vaccination^40^.

Misconception about the protective effect of smoking against COVID-19 infection might indicate that the vaccine offers little protection^41^. Several studies reported that current smokers were less likely to accept the COVID-19 vaccine^33,37^. In the current study, smokers constituted a higher proportion among the acceptance group compared to the refusal group.

In the current study, Upper Egypt governorates (Faiyum and Giza) were outpacing Lower Egypt governorates in vaccination acceptance rates. Nevertheless, the present study enrolled more urban residents (69.5%), a lagging-behind acceptance rate was encountered in urban residents (61.6% vs. 77.6%), which was in line with studies conducted by Omar and Hani and Elsayed et al. from Egypt^7,42^. The possible explanation is that, according to the Central Agency for Public Mobilization and Statistics (CAPMAS.), Upper Egypt governorates had the highest rates of illiteracy among Egyptian governorates, reaching 34% in Faiyum and 15.9% in Cairo and Giza. Individuals who obtained higher levels of education are more concerned about COVID-19 vaccine safety and potential side effects and have more tendency to refuse vaccination^43^.

Reluctance toward vaccination was associated with leading causes of refusal which had been delineated in previous studies, such as mistrust of vaccine safety due to ubiquity of negative and false information on vaccines on the internet, lack of information on different vaccines techniques, and possible side effects where reports of myocarditis and pericarditis were recorded in male adolescents and young adults after receiving Pfizer-BioNTech (BNT162b2) and Moderna mRNA-1273). In addition, reports of thrombosis and Guillain-Barré syndrome were recorded after Johnson & Johnson’s Janssen and Oxford/AstraZeneca COVID-19 vaccine (AZD1222)^44,45^.

## Conclusion

High vaccine acceptance was reported among children's guardians and elderly compared to young adults. Lower acceptance rate among HCWs was recorded. Some predictors of vaccine acceptance were male gender, rural residence, illiteracy, negative history of COVID-19 infection or contact with COVID-19 patients. Being underweight had lower odds of vaccine acceptance compared to those with normal weight. Mistrust of vaccine safety and concerns about the possible side effects were the main causes of vaccine refusal. Direct interview questionnaire allowed assessing vaccine acceptance among illiterates and elderly.

## Data Availability

The corresponding author would make the data available upon reasonable request.
